# The role of artificial intelligence in standardizing global longitudinal strain measurements in echocardiography

**DOI:** 10.1093/ehjimp/qyae130

**Published:** 2024-12-06

**Authors:** Krunoslav M Sveric, Roxana Botan, Anna Winkler, Zouhir Dindane, Ghatafan Alothman, Baris Cansiz, Jens Fassl, Michael Kaliske, Axel Linke

**Affiliations:** Department for Internal Medicine and Cardiology, Herzzentrum Dresden, Faculty of Medicine and University Hospital Carl Gustav Carus, TUD Dresden University of Technology, Fetscherstr. 76, 01307 Dresden, Germany; Department for Internal Medicine and Cardiology, Herzzentrum Dresden, Faculty of Medicine and University Hospital Carl Gustav Carus, TUD Dresden University of Technology, Fetscherstr. 76, 01307 Dresden, Germany; Department for Internal Medicine and Cardiology, Herzzentrum Dresden, Faculty of Medicine and University Hospital Carl Gustav Carus, TUD Dresden University of Technology, Fetscherstr. 76, 01307 Dresden, Germany; Department for Internal Medicine and Cardiology, Herzzentrum Dresden, Faculty of Medicine and University Hospital Carl Gustav Carus, TUD Dresden University of Technology, Fetscherstr. 76, 01307 Dresden, Germany; Department for Internal Medicine and Cardiology, Herzzentrum Dresden, Faculty of Medicine and University Hospital Carl Gustav Carus, TUD Dresden University of Technology, Fetscherstr. 76, 01307 Dresden, Germany; Institute for Structural Analysis, TUD Dresden University of Technology, 01062 Dresden, Germany; Department of Cardiac Anesthesiology, Herzzentrum Dresden, Faculty of Medicine and University Hospital Carl Gustav Carus, TUD Dresden University of Technology, Fetscherstr. 76, 01307 Dresden, Germany; Institute for Structural Analysis, TUD Dresden University of Technology, 01062 Dresden, Germany; Department for Internal Medicine and Cardiology, Herzzentrum Dresden, Faculty of Medicine and University Hospital Carl Gustav Carus, TUD Dresden University of Technology, Fetscherstr. 76, 01307 Dresden, Germany

**Keywords:** artificial intelligence, global longitudinal strain, echocardiography

## Abstract

**Aims:**

To evaluate the accuracy and feasibility of artificial intelligence (AI) in left ventricular global longitudinal strain (GLS) analysis as compared to conventional (Manual) and semi-automated (SemiAuto) method in echocardiography (Echo).

**Methods and results:**

GLS validation was performed on 550 standard Echo exams by expert cardiologists. The performance of a beginner cardiologist without experience of GLS analysis was assessed on a subset of 90 exams. The AI employs fully automated view selection, classification, endocardial border tracing, and calculation of GLS from an entire Echo exam, while SemiAuto requires manual chamber view selection, and Manual involves full user input. Interobserver agreement was assessed using the intraclass correlation coefficient (ICC) for all three methods. Agreement of measures included Pearson’s correlation (R) and Bland-Altman analysis [median bias; limits of agreement (LOA)]. With an 89% feasibility the AI showed good agreement with Manual (R = 0.92, bias = 0.7% and LOA: −3.5 to 4.8%) and with SemiAuto (r = 0.90, bias = 0.10% and LOA: −4.5 to 4%). ICCs for GLS were 1.0 for AI, 0.93 for SemiAuto, and 0.80 for Manual. After the 55th analysis, the beginner showed stable time performance with Manual (171 s), contrasting with the consistent performance of SemiAuto (85-69 s) from the beginning. The highest agreement between beginner and expert readers was achieved with AI (R = 1.00), followed by SemiAuto (R = 0.85) and Manual (R = 0.74).

**Conclusion:**

Automated GLS analysis enhances efficiency and accuracy in cardiac diagnostics, particularly for novice users. Integration of automated solutions into routine clinical practice could yield more standardized results.

## Graphical Abstract

**Graphical abstract:**
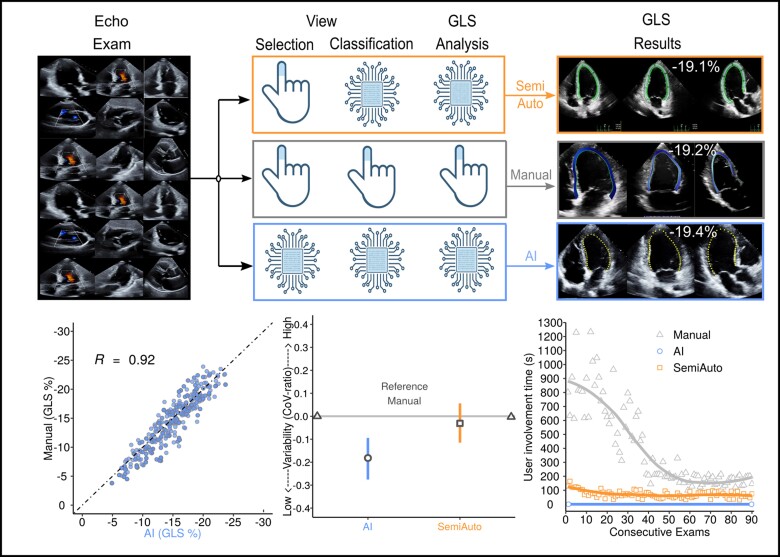
Overview of the validation of the artificial intelligence (AI)-based pipeline with automated view selection and left ventricular global longitudinal strain (GLS) measurement of echocardiography (Echo) examinations against the operator-dependent semi-automated (SemiAuto), and the conventional (Manual) method as reference.

## Introduction

Accurate assessment of left ventricular (LV) function is pivotal in diagnosing and managing cardiovascular diseases. Echocardiography (Echo) remains a key imaging modality that provides real-time, non-invasive insight into the structure and function of the heart.^1,2^ The measurement of global longitudinal strain (GLS) has emerged as a potent tool for evaluating myocardial deformation, offering additional prognostic value beyond traditional metrics such as ejection fraction (EF).^3,4^ Despite the recognized importance of GLS, its widespread adoption in routine practice has been impeded mostly by the time-intensive nature of manual analyses and the variability in measurement between operators.^5^

Artificial intelligence (AI) presents a promising solution to these challenges through fully automated view selection, classification, endocardial tracking, and GLS calculation.^6,7^ By reducing user variability, standardizing measurements, and streamlining workflows, AI has the potential to increase GLS adoption in clinical practice, benefiting both experienced practitioners and those new to the field, akin to recent advances in AI-based EF assessment.^8^ We hypothesize that a fully automated AI-based GLS measurement will perform as well as or better than operator-dependent (Manual) and semi-automated (SemiAuto) methods in terms of accuracy and efficiency. This could support the integration of GLS into routine clinical workflows, improving patient care. Our study aims to (i) validate the accuracy and variability of AI-based GLS compared to Manual and SemiAuto methods under real-world conditions, and (ii) evaluate how automated tools improve proficiency in less experienced users (*Graphical Abstract*).

## Methods

### Ethical statement

The protocol was approved by the ethics committee of the Technische Universität Dresden, Germany (#284092012 and #492112022) and conducted according to the Declaration of Helsinki. Informed consent was obtained from each participant. The study report adheres to the Guidelines for Reporting Reliability and Agreement Studies.^9^ The checklist is provided in the Supplementary data (Methods, Supplementary data online, *Table S1*).

### Subjects

Clinically indicated Echo examinations were performed by one cardiologist (K.M.S.) with 10 years of experience in Echo imaging between January 2022 and December 2023 at the Herzzentrum Dresden (Heart Centre Dresden), Technische Universität Dresden, Germany. To ensure comparability between analysis methods, we included patients aged 18 and older, in sinus rhythm, without arrhythmias or conduction disorders, with chamber views (CV) over three heartbeats, regardless of acoustic window quality. We also restricted the selection to singular examinations per patient. Duplicate apical 4-, 2-, and 3-CV were excluded from the examination, while all other loops and stills were retained as part of the original Echo examination. Furthermore, the selection process of these consecutive 550 Echo exams aimed to encompass a study cohort with diverse cardiac function.

### Echocardiographic imaging

The routine Echo examinations were performed by the same cardiologist using an EPIQ CVX device (Philips Healthcare, Andover, MA, USA) and adhered to the current guidelines.^2^ The recommended measurements of the standard functional and morphological cardiac parameters were initially performed online at the scanner. LV EF was initially assessed using the biplane Simpson method over three heartbeats, and acoustic window quality (good, fair, poor) was evaluated by the same cardiologist (K.M.S.) during the initial Echo exam.

### Global longitudinal strain analysis for validation

**Figure 1:**
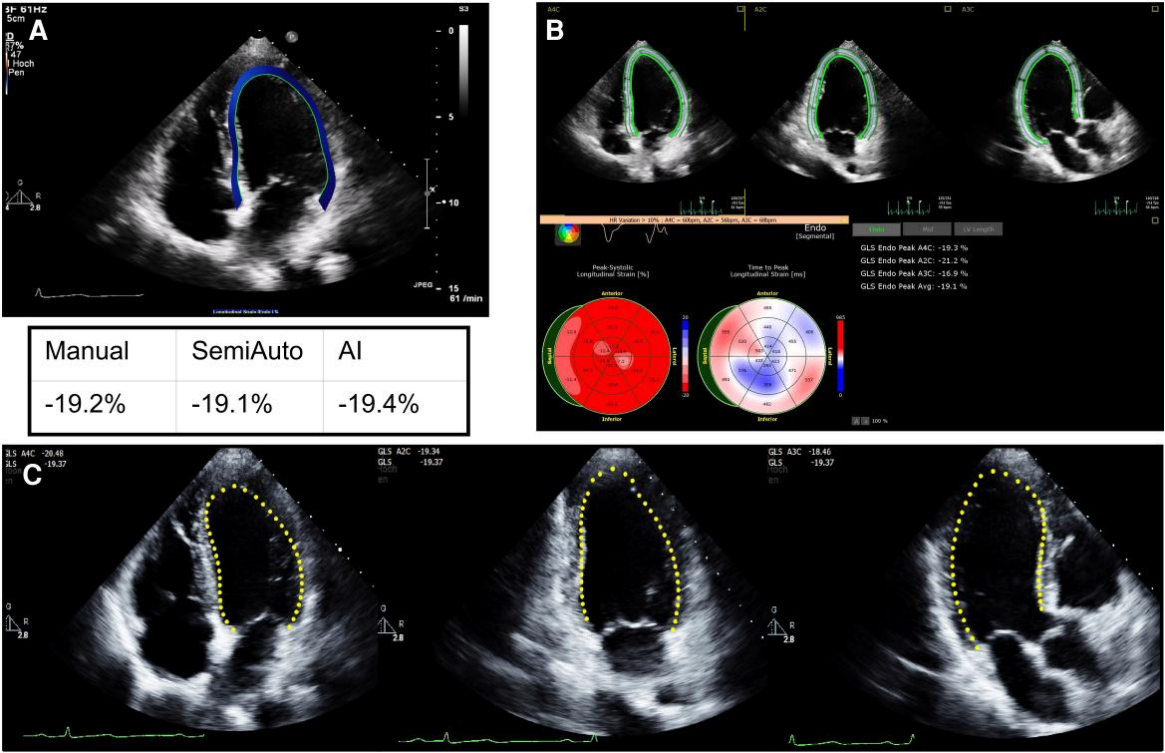
Example of left ventricular GLS results from a 47-year-old person with the Manual reference method (*A*), the SemiAuto (*B*), and the fully automated AI (*C*). AI, artificial intelligence; GLS, global longitudinal strain; SemiAuto, semi-automated; Manual, conventional operator dependant.

The GLS validation procedure started with the AI-based analysis of the Echo examination, followed by conventional GLS measurement as the reference method (Manual), and concluded with the semi-automated (SemiAuto) method of identical loops containing the CVs as follows:

First, the Echo examinations were transmitted from our in-house PACS (Picture Archiving and Communication System) to our in-house medical server, which hosted the LVivo Seamless^TM^ system (DiA Imaging Analysis, Beer-Sheva, Israel). This proprietary AI-based system automatically searches for suitable LV 4-, 2-, and 3-CVs from the entire Echo examination and, when image quality is adequate, activates the LVivoEF^TM^ module (DiA Imaging Analysis, Beer-Sheva, Israel) for fully automated detection and tracking of the LV endocardial border to calculate GLS based on all three apical views. Although the specific details of the convolutional neural network architecture were not disclosed by the company, recent studies have tested and validated this AI for GLS and LV EF assessment also across different Echo machine vendors.^10–13^ After 3–6 min, the results were ready for review either on a dedicated workstation or within the PACS (*Figure 1*). Although the system enables editing, no manual contour adjustments were made in this study, ensuring consistency and standardization in the results. The GLS results of the second heartbeat were stored on a separate server and were not accessible until the end of the validation period. Next, a cardiologist (A.W.) with board certification in Echo independently performed a conventional (Manual) LV GLS analysis on the second heartbeat, with the 2D-CPA^TM^ application (2D Cardiac Performance Analysis, TomTec Imaging System, Unterschleissheim, Munich, Germany) as the reference method.^14^ After a blanking period of three weeks, the same cardiologist subsequently analysed identical CV loops using the TomTec AutoLV^TM^ software on the second heartbeat, hence after called semi-automated (SemiAuto). This SemiAuto system requires manual selection of the three apical CVs, but it performs automated view classification, endocardial border delineation, and tracking, as well as calculating GLS values.^15^ Additionally, this application allows for manual adjustment of the results, a process performed in 38% (*n* = 210) of all 550 cases.

### Reliability measurements of global longitudinal strain

To assess interobserver reliability, an independent board-certified cardiologist (R.B.) re-assessed GLS in a random subset of 38 patients using the AI (i.e. uploading the exams), SemiAuto, and Manual methods. The techniques were performed as described above.

### Performance assessment of the beginner

We aimed to assess agreement between GLS measurements conducted by a beginner cardiologist (G.A.) and the initial results. The beginner underwent training, which involved 10 exams (not included in the subsequent analysis) using the AI, SemiAuto, and Manual methods under the supervision of one experienced cardiologist (K.M.S.), who was not involved in the GLS validation assessment. Following this training, 90 Echo exams were randomly selected from the final cohort. The beginner analysed these exams independently and sequentially, using AI (i.e. uploading the exams), followed by the SemiAuto and Manual methods; each method was performed after 3-week intervals and in the same sequence of exams to ensure comparability. GLS measurements were performed as described above, and the results were stored separately and inaccessible to both the beginner and experts. The time spent by the beginner on CV selection, setting endocardial border points (systole and diastole), and tracking adjustments was measured independently.

### Statistics

Values are reported as medians with interquartile ranges (25th; 75th percentiles) or frequencies with percentages. Pearson’s correlation coefficients (R) were calculated, and Bland-Altman analyses were performed with non-parametric limits of agreement (LOA) due to non-normal inter-method differences.^16^ Subgroup analyses were conducted for LV EF (≤35%, 36–49%, ≥50%), acoustic window quality (good, fair, poor), and body mass index (BMI > or <30 kg/m²). Measurement bias between AI and SemiAuto vs. the Manual method was assessed using multivariable least squares regression, with LV EF, acoustic window, BMI, sex, and age as confounders. Continuous variables were fitted with restricted cubic splines (four knots) to capture non-linear relationships and were presented as adjusted marginal effects with 95% credibility intervals (CI).^17^ Model assumptions and diagnostics were visually inspected. A cut-off value of −16% was used to define abnormal GLS.^18^ Receiver operating characteristic curve analysis, was used to assess diagnostic performance, with the area under the curve (AUC) indicating overall accuracy. Sensitivity, specificity, positive predictive value (PPV), and negative predictive value (NPV) were calculated. The coefficient of variation (CoV) ratio with 95% CI was calculated to compare GLS variability across methods. A ratio < 0 indicates lower variability with AI or SemiAuto. Interobserver agreement was evaluated using the intraclass correlation coefficient (ICC).^19^ The 95% CI for the bias regression and CoV-ratio, as well as the median of the subgroup AUCs, were derived using the bootstrapping method (10 000 repetitions). User involvement time during GLS measurements by the beginner was modelled with non-linear regression using an inverse sigmoid function for learning curve analysis. The inflection point was derived from the sigmoid formula.^20^⁠ Learning rate (i.e. user involvement time, absolute GLS difference |Δ| between beginner and expert, and ICC) was assessed for the first 30, 31–60, and 61–90 Echo exams, with the last third used for plateau calculation (see Supplementary data, Methods). A sample size of 133 (power = 0.90; alpha = 0.05) was calculated based on prior studies,^6,15^ with a total of 450 patients considered sufficient for analysis among three subgroups.^21^ Statistical significance was defined as a two-tailed *P*-value <0.05. All analyses were conducted using R software (version 3.0.2, The R Foundation for Statistical Computing, Vienna, Austria).

## Results

Patients were aged between 18 and 91 years, with a predominance of male sex (*n* = 286, 59%). Twenty-four percent (*n* = 117) were obese, and the cohort exhibited a wide range of LV EF, from 13% to 70%. Further characteristics of the cohort are shown in *Table 1*. The GLS measurements on 4-, 2-, and 3-CV were performed in all 550 Echo exams using both the Manual and the SemiAuto methods. The AI method was feasible in 89% (*n* = 489) with a 100% correct CV classification, forming the final cohort. A total of 11% (*n* = 61) of cases were rejected by the AI system due to poor image quality or inadequate acoustic windows, as illustrated in the Supplementary data (Results, Supplementary data online, *Figure S1*).

**Table 1:** Characteristics of the studied patients (*n* = 489)

Demographics	
Age (years)	69 (58;80)
Male sex	288 (59)
Clinical characteristics	
Body mass index (kg/m²)	25.9 (24.3;29.6)
Heart rate (beats/min)	67 (64;77)
Indication for echocardiography	
Coronary artery disease	191 (39)
Valvular heart disease	200 (41)
Non-ischaemic cardiomyopathies	73 (15)
Other	25 (5)
LV EF category	
≤35%	137 (28)
36% to 49%	142 (29)
≥50%	210 (43)
Acoustic window quality	
Good	254 (52)
Fair	171 (35)
Poor	64 (13)

Values are reported as *n* (%) or median (interquartile range).

LV EF, left ventricular ejection fraction.

### Comparison of global longitudinal strain measurements among the methods

**Figure 2:**
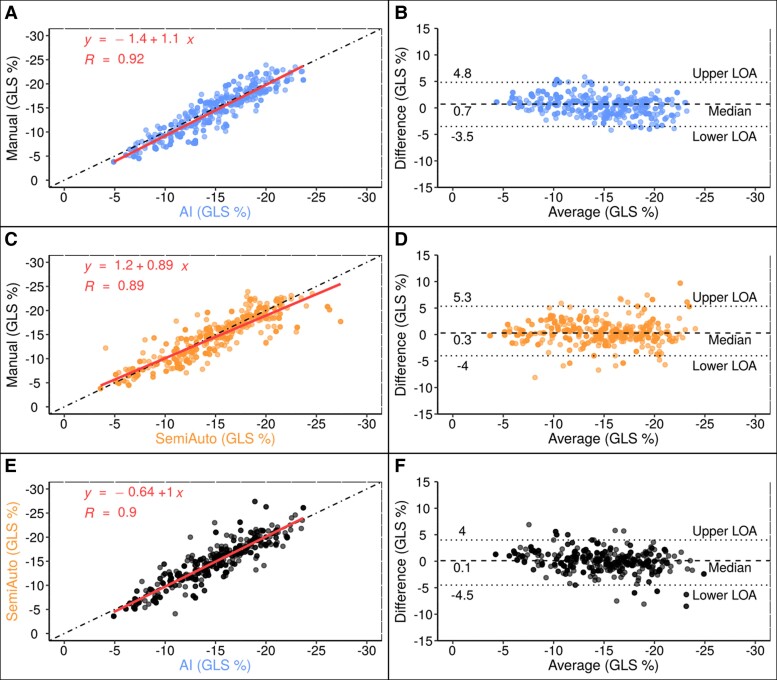
Comparison of GLS values from AI, SemiAuto, and Manual as correlation plots (*A*, *C*, and *E*) and Bland-Altman plots (*B*, *D*, and *F*). LOA, limits of agreement; R, Pearson’s correlation coefficient, otherwise as in *Figure 1*.

The median GLS value in the final cohort was −14.9% (−17.6; −11.7) for the AI, −14.6% (−17.7; −10.4) for the Manual, and −14.5% (−18.1; −11.5) for the SemiAuto method. GLS correlations were excellent between AI and Manual methods (*r* = 0.92), SemiAuto and Manual (*r* = 0.89), and AI and SemiAuto (*r* = 0.90). Both, AI and SemiAuto showed small biases against the Manual method, with AI having narrower LOA than SemiAuto. Full details are provided in *Figure 2*. A summary of the descriptive statistics and the parameters from Bland-Altman analysis is provided in Supplementary data (Results, Supplementary data online, *Tables S2* and *S3*).

### Source of bias and variability of measurements between methods

**Figure 3:**
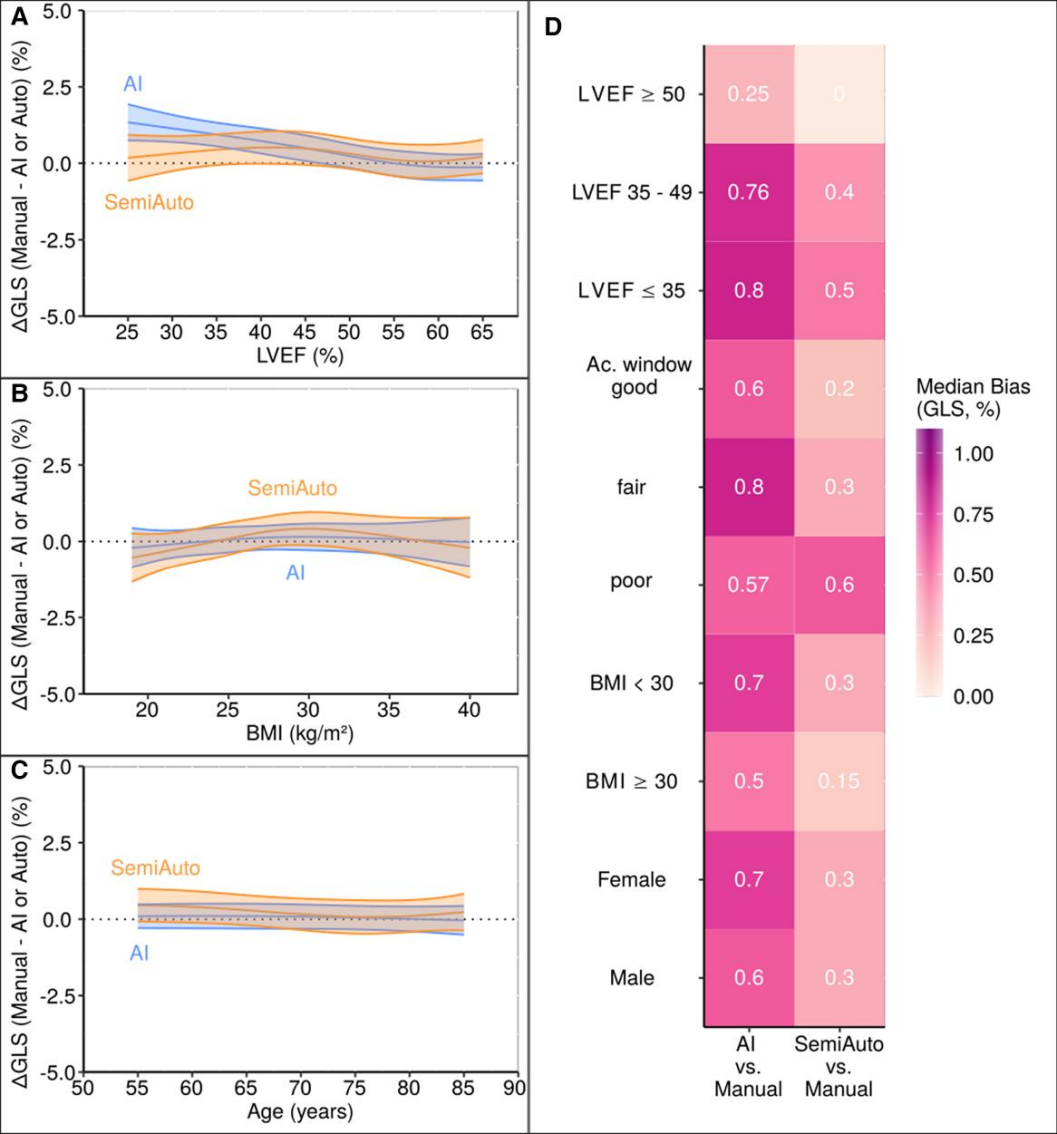
Marginal bias plots (*A*, *B*, and *C*) of GLS from AI and SemiAuto against Manual as the reference for LVEF, BMI, and patients’ age for the entire cohort. Heat-map plot (*D*) of median GLS biases in subgroups. LVEF, left ventricular ejection fraction; Ac., acoustic; BMI, body mass index, otherwise as in *Figure 1*.

**Figure 4:**
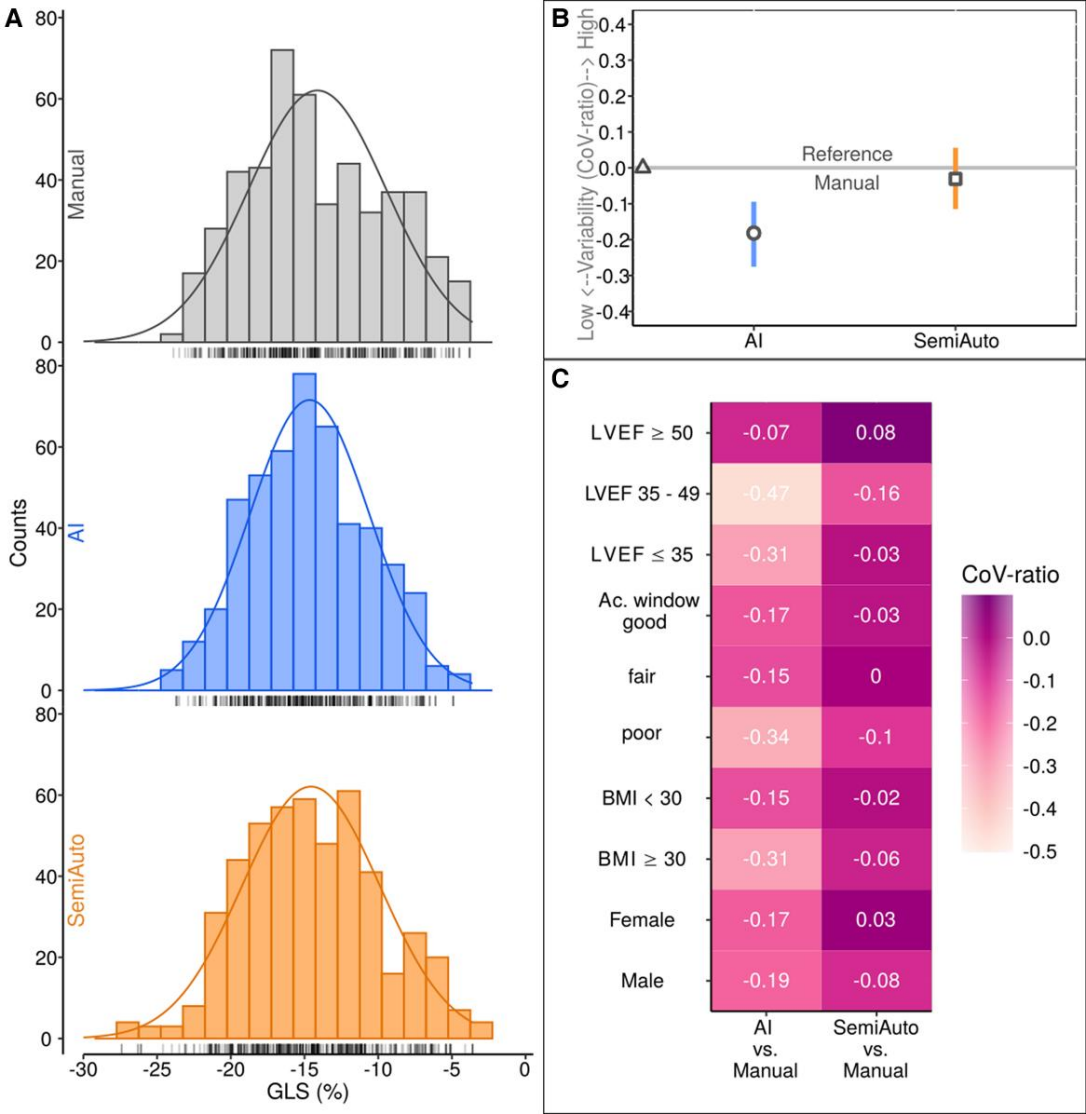
Distribution of GLS values for manual, AI and SemiAuto as histogram plots (*A*) and GLS measurement variability of AI and SemiAuto against the reference manual (*B*) in the entire cohort. CoV-ratio analysis for subgroups (*C*). CoV, coefficient of variation, otherwise as in *Figures 1* and *3*.

Multivariable bias regression showed minimal GLS differences between AI, SemiAuto, and Manual methods across the entire cohort (*Figure 3A*). Subgroup analysis by LV EF, acoustic window quality, BMI, and sex revealed indeed only neglectable differences (*Figure 3B*). The AI method had the lowest dispersion and a more symmetrical distribution of GLS values compared to Manual and SemiAuto methods (*Figure 4A*). The CoV-ratio for GLS was lower for AI than SemiAuto, using Manual as the reference, in both the full cohort and subgroups (*Figure 4B* and *C*). See Supplementary data (Results, Supplementary data online, *Figure S2*) for details.

### Diagnostic performance in detecting abnormal GLS

**Figure 5:**
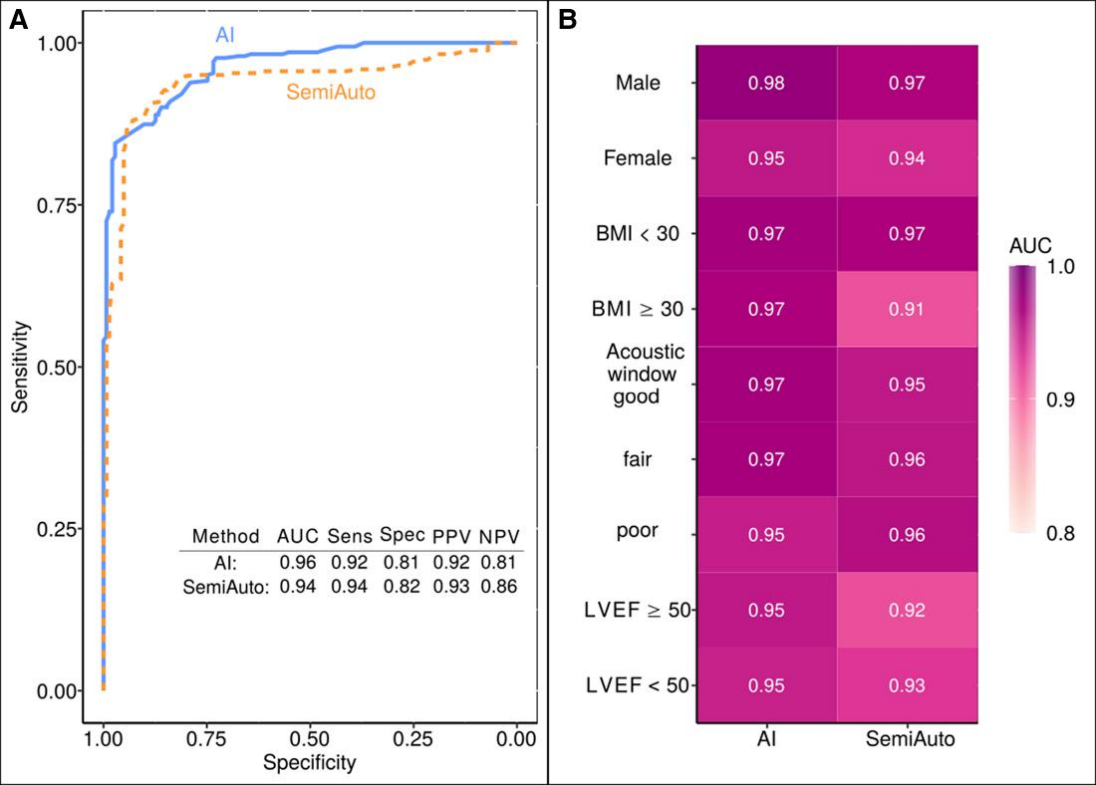
Detection accuracy of abnormal GLS (> −16%) by AI and SemiAuto for the entire cohort (*A*) and for the subgroups (*B*). AUC, area under the curve; Sens, sensitivity, Spec, specificity, PPV, positive predictive value, NPV, negative predictive value, otherwise as in *Figures 1* and *3*.

Both, the AI and SemiAuto method showed similar high diagnostic accuracy in the detection of an abnormal GLS value as determined by the reference method Manual not only for the entire cohort but also for subgroups for LV EF classes, acoustic window quality, obesity, and sex (*Figure 5*). A scenario-based sensitivity analysis showed that the automated component of the SemiAuto method, without manual adjustments, produced median GLS values comparable to the AI method (−14.1% vs. −14.7%) with identical AUC values for detecting an abnormal GLS (0.98). See Supplementary Data (Results, Supplementary data online, *Figure S3*).

### Reliability of repeated measurements

Interobserver ICC for GLS using the AI method was 1.00. The corresponding ICCs by the expert cardiologists were 0.93 for the SemiAuto and 0.80 for the Manual method. Additionally, the correct reclassification rate of the 4-, 3-, and 2-CVs by the AI method was 100%.

### Beginner’s performance of GLS measurements

**Figure 6:**
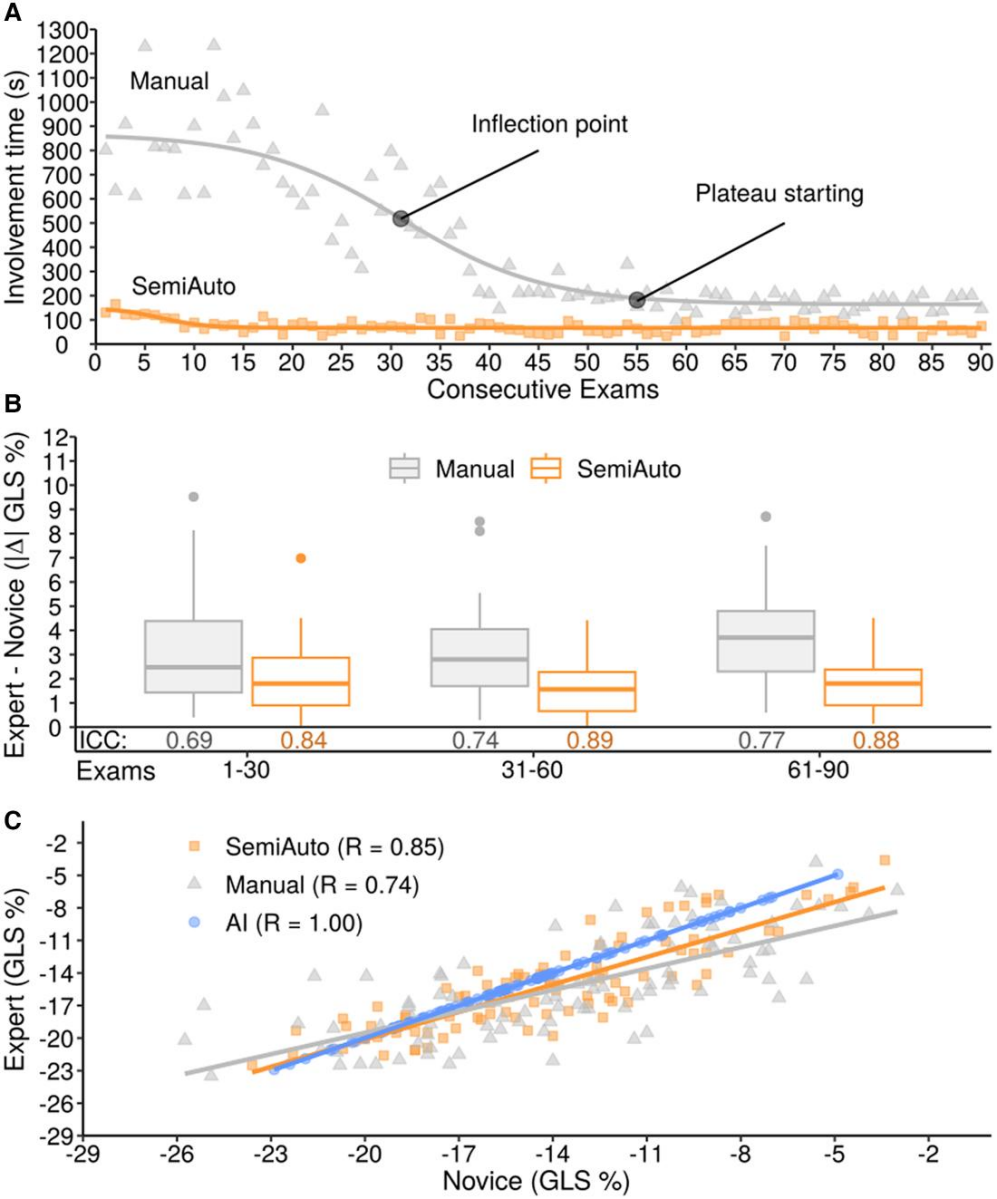
Analysis involvement time for GLS measurement by a beginner on consecutive exams for manual and SemiAuto method (*A*). Mean absolute difference as box plots and ICC for SemiAuto and Manual GLS between the beginner and expert for each tercile of exams (*B*). Comparison of GLS values between beginner and Expert for SemiAuto, Manual, and AI as correlation plot (*C*). ICC, intraclass correlation coefficient, otherwise as in *Figures 1*, *2*, and *3*.

The beginner’s involvement time for GLS analysis with the Manual method decreased from a mean of 749 s in the first tercile to 292 s in the second tercile and to 171 s in the third tercile. The inflection point occurred at exam 31, with a stable plateau reached by exam 55 (*Figure 6A*). In contrast, the beginner’s involvement time with the SemiAuto method remained stable, starting at a mean of 85 s in the first tercile, 66 s in the second, and 69 s in the third. The median absolute difference in GLS values between the beginner and expert remained higher with the Manual method across all terciles (2.6%, 2.8%, and 3.7%) compared to the SemiAuto method (1.8%, 1.6%, and 1.8%) (*Figure 6B*). The beginner and expert cardiologists achieved higher correlation for GLS measurements with the SemiAuto method compared to the Manual method (*Figure 6C*).

## Discussion

In the present study, we evaluated the good feasibility and high accuracy of a fully automated AI-based GLS analysis application in a large patient cohort with a broad range of LV function. Integration of LV view selection and GLS quantification within a unified workflow of this AI not only outperformed interobserver agreement of the conventional method, but also the SemiAuto method. In addition, these two automated methods were particularly beneficial for beginners, highlighting their potential for clinical practice. Key results are summarized in the *Graphical Abstract.*

Echo represents the first diagnostic imaging method in screening for impaired LV function, and evidence supports the prognostic significance of GLS, suggesting its superiority over EF in predicting major adverse cardiac events.^3,4,22^ Hence, in the clinical setting, accurate and dependable assessment of LV GLS through Echo is pivotal for guiding clinical decision-making. To our knowledge, our study is the first to compare GLS measurements using three different methods: fully automated AI, SemiAuto, and the Manual method. Using this approach, we gained a more comprehensive understanding of the clinical utility of this automated AI in different patient characteristics, including a wide range of LV EF, image quality and degree of obesity (*Figures 2, 3*, and *5*). Our results are consistent with recent studies comparing other AI-based systems with either the Manual or the SemiAuto method only, reporting correlations ranging from 0.54 to 0.93 and absolute biases between 0.3% and 3.3%.^6,7,23–25^ One might ask why we did not use clinical diagnoses like coronary artery disease or valvular heart disease for subgroup analysis. This was intentional, as factors such as sex, image quality, and obesity pose more immediate challenges in Echo and subsequent GLS analysis. A stratification by clinical diagnosis is less practical in this validation setting due to overlap of LV function between clinical groups. As standard GLS assessment is highly operator-dependent (interobserver ICC 0.80), AI-based methods—whether fully automated or semi-automated—significantly reduce measurement variability (interobserver ICC 1.0 and 0.93), consistent with previous reports.^26–28^ Therefore, automated methods offer a viable solution for consistent reporting across users, improving clinical reliability and accessibility for daily use.

Indeed, several guidelines recommend the use of GLS to quantify LV function beyond EF,^29,30^ but its routine clinical use remains limited, due to time constraints and hesitation to perform the analysis.^5,31^ An application that reduces reluctance to measure GLS in daily practice and minimizes measurement variability would be highly desirable, especially for beginner cardiologists or infrequent users. A recent study recommends analysing at least 50 exams to achieve proficiency in standard GLS analysis.^32^ This aligns with our observation, where a beginner cardiologist, familiar with Echo but new to strain imaging, needed 31 exams to improve analysis time, reaching stability and agreement with an expert by the 55th exam (*Figure 6*). In contrast, the AI or SemiAuto user achieved immediate success, while the Manual method relies entirely on operator expertise.

As our research contributes to a broader transformation evolving in the field of Echo, AI has the potential to augment expert knowledge as a ‘feedback’ tool, especially in settings with limited clinical expertise. This applies not only to strain analysis but also extends to LV EF calculation, as well as guidance, support, and training in Echo acquisition, benefiting not only future cardiologists but the wider clinical team.^33–37^

By integrating AI, our study demonstrates that abnormal GLS is detected as accurately as with the conventional method (*Figure 5*), potentially lowering the threshold for access to advanced cardiac imaging in the future. The same holds true for patient evaluations in other settings, including auscultation, and electrocardiogram assessment, enhancing precision and enabling personalized care or real-time monitoring.^38^ However, this also raises ethical and data protection concerns, particularly with the increasing use of wearables. With any technological advancement, physicians—particularly those in training—must remain vigilant to develop an over-reliance on AI-based tools.^38^ As shown in our example (see Supplementary Data, Supplementary data online, *Figure S1*), the SemiAuto system completed GLS analysis on suboptimal loops, whereas the fully automated AI system rejected them. In such instances, clinicians must manually assess strain. Neither method currently supports contrast-enhanced GLS analysis, sometimes necessitating referral to alternative imaging modalities. Given AI’s sensitivity to image quality, further developments are needed to enhance robustness across diverse clinical settings.

Arrhythmias, such as atrial fibrillation, affect the accuracy of GLS and LV EF measurements. In our study, the AI systems used the second heartbeat by default, which may limit applicability and clinical decision-making in these patients. To overcome this, it is advisable to acquire Echo loops during representative cardiac phases. If this is not possible, averaging more than three beats or using the index-beat approach is recommended by applying conventional methods.^39^ Future AI validations should include patients with arrhythmias, emphasizing the importance of clinician training for effective integration of AI into practice while maintaining traditional expertise. AI-based approaches hold clinical promise, but software, maintenance, and hardware costs must be balanced against reimbursement, which varies by institution and country.

Therefore, it is crucial to rigorously evaluate AI systems to ensure reliable and accurate outputs, as performed in our study. This is especially relevant for proprietary systems, where details of their architecture and learning processes are not fully disclosed. Future long-term, multi-centre studies should compare different AI applications across different Echo vendors to assess how AI might influence thresholds for intervention or monitoring in various settings. This could offer new perspectives for clinical guidelines.

### Limitations

This study had several limitations, despite a large cohort of patients with a broad range of LV function. The single-centre nature of this study may limit generalisability of findings. The proprietary nature of both AI algorithms restricts full transparency, emphasizing the need for thorough testing, as we have done with this study. Although speckle tracking methods have been standardized across vendors,^40^ our validation of the novel AI was based on a single Echo machine. However, the AI’s vendor-neutral capability has recently been tested with other manufacturers not included in this study.^10–12^ Further research is needed to confirm these results in specific groups, like patients with atrial fibrillation. Recently, we validated LV EF measurements from this AI in these patients, showing good agreement with conventional assessments.^13^ Last, the lack of follow-up data limits the assessment of the clinical impact and cost-effectiveness of this AI-based method, particularly in outpatient or resource-limited settings.

## Conclusion

The AI method demonstrated high feasibility and accuracy, providing reliable GLS measurements with consistent performance across different user levels, coupled with minimal variability and high inter-method agreement. Our findings suggest that incorporating an automated system has the potential to improve and enable analysis of GLS in a broad range of patients.

## Supplementary Material

qyae130_Supplementary_Data

## Data Availability

Data are available from the authors upon reasonable request and with permission of the Ethics committee of the Technische Universität Dresden, Dresden, Germany.

## Lead author biography

Dr Krunoslav Michael Sveric is a senior cardiologist at the Heart Centre Dresden, Faculty of Medicine Carl Gustav Carus at the Technical University of Dresden (Germany), where he heads the Echocardiography Laboratory. His specialization is in non-invasive advanced cardiovascular imaging, with a particular focus on 3D echocardiography and the integration of artificial intelligence to enhance patient diagnostics. Dr Sveric is a prolific contributor to numerous peer-reviewed publications and congresses both at national and European level. In addition to his involvement in clinical trials, he is deeply committed to teaching and mentoring young researchers and students at the Technical University of Dresden, thereby fostering the next generation of cardiovascular specialists.
