# Supplementary material for: The role of artificial intelligence in standardizing global longitudinal strain measurements in echocardiography
Source: Eur Heart J Imaging Methods Pract. 2024 Dec 6;2(4):qyae130. doi: 10.1093/ehjimp/qyae130 (PMC11660427; doi:10.1093/ehjimp/qyae130)
Supplement: qyae130_Supplementary_Data [file qyae130_supplementary_data.docx]

Appendix / Supplemental Data

**The Role of Artificial Intelligence in Standardising Global Longitudinal Strain Measurements in Echocardiography**

Krunoslav M. Sveric^a^,MD; Roxana Botan^a^,MD; Anna Winkler^a^,MD; Zouhir Dindane^a^,MD; Ghatafan Alothman^a^,MD; Baris Cansiz^b^,PhD; Jens Fassl^c^,MD,PhD; Michael Kaliske^b^,PHD; Axel Linke^a^,MD

^a^ Department for Internal Medicine and Cardiology, Herzzentrum Dresden, Faculty of Medicine and University Hospital Carl Gustav Carus, TUD Dresden University of Technology, Fetscherstr. 76, 01307, Dresden, Germany

^b^ Institute for Structural Analysis, TUD Dresden University of Technology, 01062 Dresden, Germany

^c^ Department of Cardiac Anesthesiology, Herzzentrum Dresden, Faculty of Medicine and University Hospital Carl Gustav Carus, TUD Dresden University of Technology, Fetscherstr. 76, 01307, Dresden, Germany

Table of Contents

[Methods 2](#__RefHeading___Toc14113_2063398854)

[Reporting checklist (Table S1) 2](#__RefHeading___Toc14115_2063398854)

[Statistical procedure 3](#__RefHeading___Toc570_4145333441)

[Learning Curve Analysis 3](#__RefHeading___Toc1130_343356494)

[Results 4](#__RefHeading___Toc9033_1487724923)

[Examples of Analysis Rejection by AI (Figure S1) 4](#__RefHeading___Toc8239_343356494)

[Summary of descriptive statistics (Table S2) 5](#__RefHeading___Toc8398_343356494)

[Summary of comparison statistics (Table S3) 5](#__RefHeading___Toc9041_1487724923)

[Variation Analysis of GLS results among the Methods (Figure S2) 6](#__RefHeading___Toc8541_1312825119)

[Scenario based Sensitivity Analysis of the SemiAuto Method (Figure S3) 7](#__RefHeading___Toc4319_1562471725)

# Methods

## Reporting checklist (Table S1)

| **Table S1: Checklist - Guidelines for Reporting Reliability and Agreement Studies (GRRAS)** in: *Kottner J., Audigé L., Brorson S., et al. Guidelines for Reporting Reliability and Agreement Studies (GRRAS) were proposed. J Clin Epidemiol 2011;64(1):96–106. Doi: 10.1016/j.jclinepi.2010.03.002.* | | | |
| --- | --- | --- | --- |
| Section | Item # | Checklist item | Reported on manuscript page # |
| Titel/Abstract | 1 | Identify in title or abstract that interrater/intrarater  reliability or agreement was investigated. | abstract |
| Introduction | 2 | Name and describe the diagnostic or measurement  device of interest explicitly. | 1 |
|  | 3 | Specify the subject population of interest. | 1 |
|  | 4 | Specify the rater population of interest (if applicable). | 1 |
|  | 5 | Describe what is already known about reliability and agreement and provide a rationale for the study (if applicable). | 1 |
| Methods | 6 | Explain how the sample size was chosen. State the  determined number of raters, subjects/objects, and  replicate observations. | 2,5 |
|  | 7 | Describe the sampling method. | 2 |
|  | 8 | Describe the measurement/rating process (e.g. time  interval between repeated measurements, availability of clinical information, blinding). | 3,4 |
|  | 9 | State whether measurements/ratings were conducted independently. | 2 to 4 |
|  | 10 | Describe the statistical analysis. | 5 |
| Results | 11 | State the actual number of raters and subjects/objects which were included and the number of replicate observations which were conducted. | 6 |
|  | 12 | Describe the sample characteristics of raters and  subjects (e.g. training, experience). | 6,7 |
|  | 13 | Report estimates of reliability and agreement including measures of statistical uncertainty. | 5,6,7 |
| Discussion | 14 | Discuss the practical relevance of results. | 9 |
| Auxiliary  material | 15 | Provide detailed results if possible (e.g. online). | Tables, Figures and Supplemental data |

## Statistical procedure

### Learning Curve Analysis

For the learning curve analysis of beginner involvement time in GLS analysis using the standard Manual method, we applied a sigmoid function

$y=c+\frac{\left( d-c \right)}{1+e^{\left( -a\left( x-b \right) \right)}}$ ,

where y represents the involvement time, x is the number of consecutive exams, and b, c, and d are constants, with x=b being the inflection point.

The sigmoid function forms a logistic curve, a mathematical model that describes an "S-shaped" curve. This model is particularly suited to learning processes where improvement occurs in distinct phases: an initial period of slow progress, followed by rapid learning, and concluding with a plateau as mastery or saturation is reached. This characteristic makes the sigmoid function a flexible, non-linear model for learning curves, especially when the process exhibits finite capacity and structured phases.

Specifically, non-linear regression analysis with iterative algorithm optimisation based on the sigmoid function was employed. The fitted results produced a high pseudo R-squared of 0.84, a root mean square error (RMSE) of 123.95, and an Akaike Information Criterion (AIC) of 875.57. These metrics were superior to those obtained using the more commonly employed power-law function

$y=AX^{b}$,

where y is the involvement time, x is the number of consecutive exams, and A and b are constants, with b representing the exponent that defines how y scales with changes in x. The power-law function resulted in a low pseudo R-squared of 0.47, an RMSE of 214.23, and an AIC of 970.08 in this study.

To calculate the point where the plateau of the sigmoid curve begins, the mean and standard deviation of involvement time in the last tercile of exams (i.e., exams 61 to 90, see main text, Figure 6C) were used to estimate where the sigmoid function reaches its relative minimum value within a certain margin (epsilon = standard deviation of involvement time in the third tercile). This approach was necessary because the mathematical minimum/maximum of the sigmoid function is an asymptote, meaning it theoretically "never" reaches the exact minimum or maximum value (±∞).

# Results

## **Examples of Analysis Rejection by AI (Figure S1)**


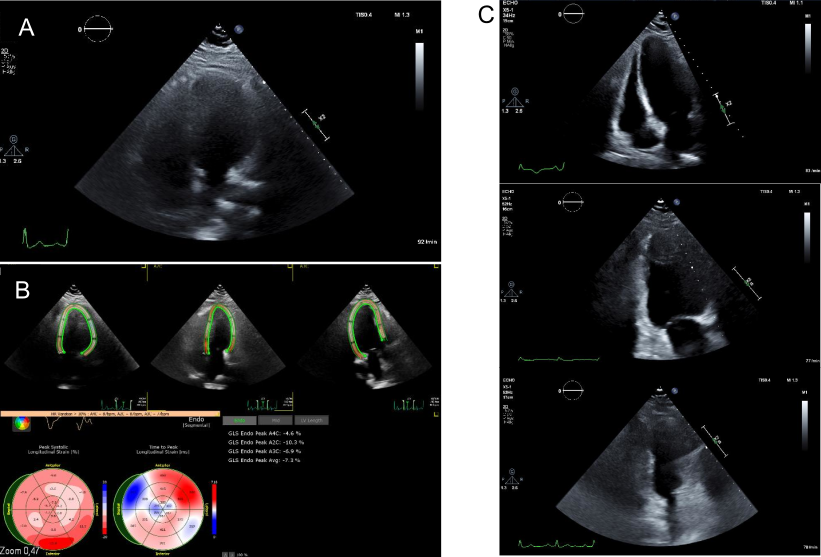


Figure S1: Examples of analysis rejection by the fully automated AI-based method.
(A) The 4-chamber view was rejected by the AI due to impaired delineation of the left ventricular endocardium, while the semi-automated (SemiAuto) method performed GLS analysis after manual selection of the loops (B). (C ) Not only an impaired acoustic window, due to missing wall segments (bottom panel) or shadowing of the anterior wall (mid panel), but also the inappropriate acquisition of a 4-chamber view centered primarily on the right ventricle (top panel) were causes of analysis rejection by the AI. Of note, the SemiAuto performed the GLS analysis.

## Summary of descriptive statistics (Table S2)

| 1. **Table S2:** Comparison of global longitudinal strain (GLS) values of the main manuscript for the reference method (Manual), artificial-intelligence (AI) based method, the semi-automated (Semi-Auto) and the automated (Auto) method without user adjustment of tracings. Values are shown in % as units. | | | | | |
| --- | --- | --- | --- | --- | --- |
|  | 1. Mean | 1. Median | 1. Standard deviation | 1. Interquartile range 2. (0.25 percentile;0.75 percentile) | 1. Total range |
| 1. Reference: Manual | 1. -14.1 | 1. -14.6 | 1. 4.7 | 1. -17.7 to -10.4 | 1. -23.9 to -3.8 |
| 1. AI | 1. -14.7 | 1. -14.9 | 1. 4.1 | 1. -17.6 to -11.7 | 1. -23.7 to -4.9 |
| 1. Semi-Auto | 1. -14.6 | 1. -14.5 | 1. 4.7 | 1. -18.1 to -11.5 | 1. -27.4 to -3.6 |

## **Summary of comparison statistics (Table S3)**

| **Table S3:** Agreement for LV GLS values based on correlations and Bland-Altman analyses in Echo (n = 489) | | | | |
| --- | --- | --- | --- | --- |
|  | R | Median Bias | Lower LOA | Upper LOA |
| Methods: |  |  |  |  |
| AI vs. Manual | 0.92 | 0.7 | -3.5 | 4.8 |
| SemiAuto vs. Manual | 0.89 | 0.3 | -4.0 | 5.3 |
| AI vs. SemiAuto | 0.90 | 0.1 | -4.5 | 4.0 |
| Bias and limits of agreement are expressed in strain units.  GLS= global longitudinal strain; AI = artificial intelligence; SemiAuto = semi-automated; Echo = echocardiography; R = Pearson correlation coefficient; LV = left ventricular; LOA = limits of agreement. | | | | |

## **Variation Analysis of GLS results among the Methods (Figure S2)**


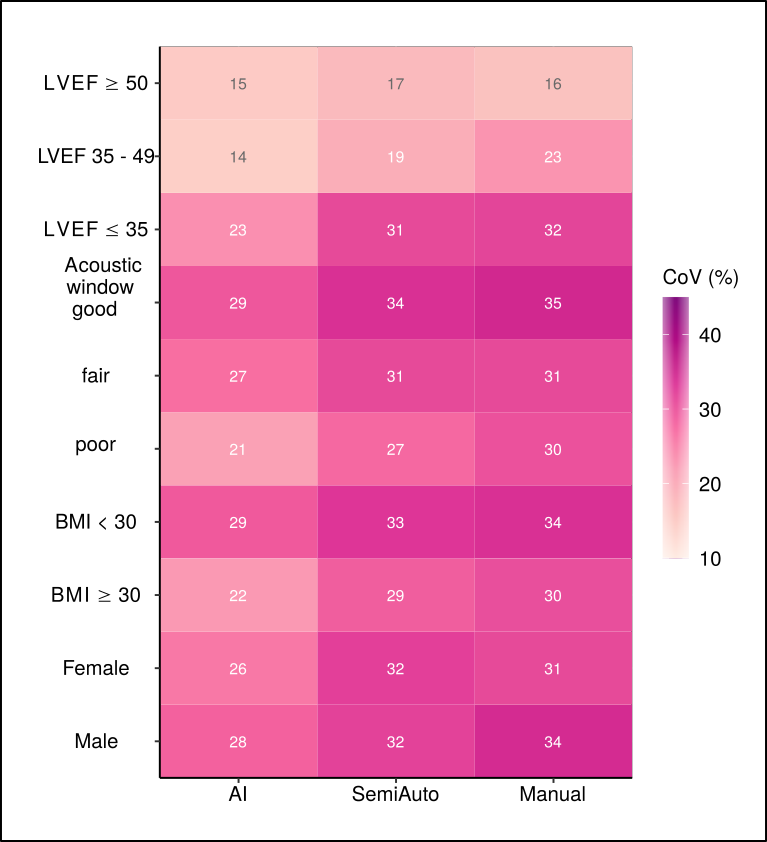


Figure S2: Coefficient of Variation (CoV) of GLS values among measurement methods for subgroups for the calculation of the CoV-ratio in Figure 4 of the main manuscript text.

CoV values for the fully automated AI-based method were lower in all subgroups as there are sex, body mass index (BMI), acoustic window and left ventricular ejection fraction (LV EF) classes, except for cases with LV EF < 35%. In this subgroup, CoV values were almost identical, thus providing a bootstrapping based CoV-ratio between -0.07 and 0.08 as depicted in Figure 4 of the main manuscript.

## **Scenario based Sensitivity Analysis of the SemiAuto Method (Figure S3)**

## **
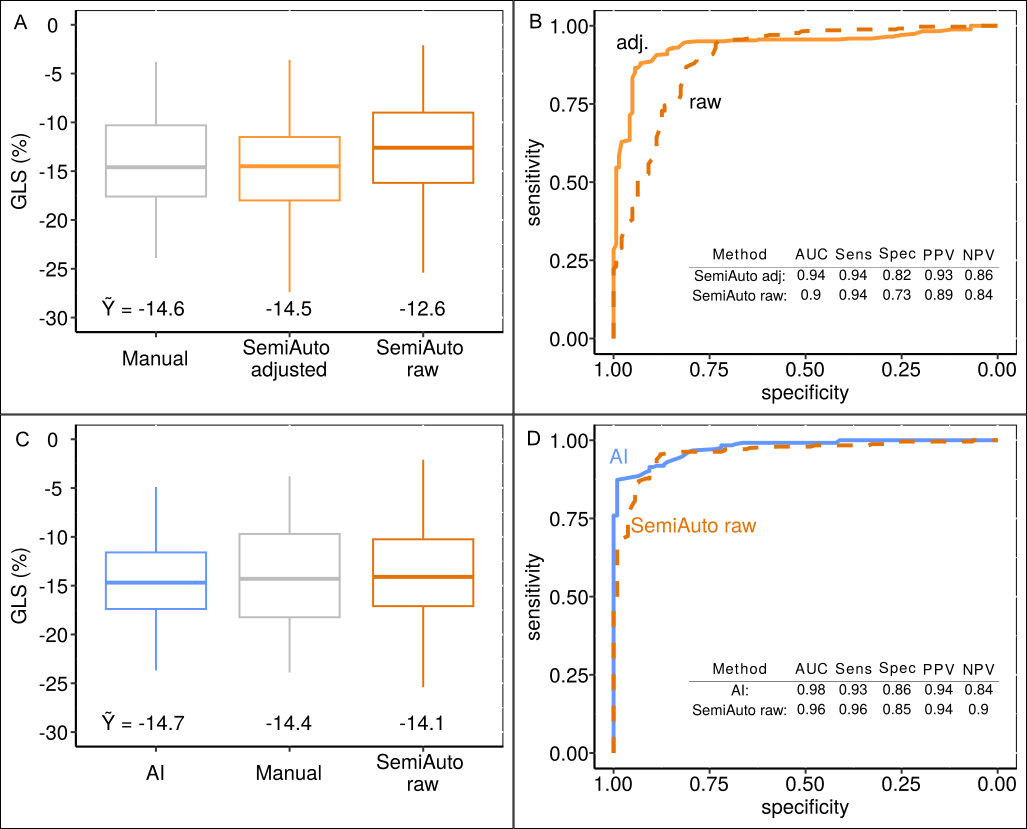
**

Figure S3: Scenario-based sensitivity analysis of the automated component of SemiAuto method.

(A) Comparison of GLS values obtained by the Manual method, SemiAuto method with operator dependent adjustments of the endocardial border delineation and without adjustments (i.e. raw) in all cases of the final cohort. Unadjusted (i.e. raw) SemiAuto results of median (Ỹ) GLS values were smaller than compared to Manual method or the adjusted SemiAuto method.

(B)Thus, AUC values and concomitantly the diagnostic performance metrics for the detection of an abnormal GLS as determined by the reference method Manual, were smaller.

(C ) However, excluding all cases (25 %, n = 122) with a necessary operator dependent adjustment of endocardial GLS border results from SemiAuto method revealed similar median (Ỹ) GLS values between the AI-based fully automated method and the reference method Manual.

(D) The AUC for the detection of an abnormal GLS, as defined by the Manual method, was identical between the automated component of SemiAuto and the fully-automated AI methods (0.98 vs. 0.98). Sensitivity (Sens), Specificity (Spec), positive predictive value (PPV), and negative predictive value (NPV) were almost identical.
